# Dramatic reduction in hepatitis B through school-based immunization without a routine infant program in a low endemicity region

**DOI:** 10.1186/s12879-015-0979-8

**Published:** 2015-06-12

**Authors:** Teegwendé Valérie Porgo, Vladimir Gilca, Gaston De Serres, Michèle Tremblay, Danuta Skowronski

**Affiliations:** Centre hospitalier universitaire de Québec, Université Laval, Québec, Canada; Université Laval, Institut National de Santé Publique du Québec, 2400 d’Estmauville, Beauport, Québec, Qc G1E7G9 Canada; Direction Régionale de Santé Publique de Montréal, Montréal, Canada; British Columbia Center for Diseases Control, Vancouver, Canada

## Abstract

**Background:**

Hepatitis B (HB) prevention in the low-endemicity province of Quebec Canada, (population: ~8.2 million; birth cohort ~85,000/year), includes two decades of pre-adolescent school-based immunization, as well as catch-up immunization for those born since 1983 and pre-natal maternal HBsAg screening. To estimate the potential added benefit of routine infant HB immunization, notifiable disease reports were analyzed (1990–2013). Clinical and demographic information about cases was retrieved from standard questionnaires used by local public health units to investigate HB cases.

**Methods:**

The Quebec provincial registry of notifiable diseases was used to identify confirmed HB cases reported between 1990 and 2013. Clinical and demographic information on cases was retrieved from the standard questionnaires used by local public health units to investigate reported HB cases.

**Results:**

Between 1990–2013, acute-HB incidence per 100,000 population decreased by 97 % from 6.5 to 0.2. Compared to 1990, incidence fell from 0.6 to zero since 2010 among children ≤9 years of age (yoa), from 3.2 to zero since 2007 in those 10–19 yoa, and from 15 to zero in 2013 among adults 20–29 yoa, previously the age group of highest incidence (all p < 0.0001).

During the same period, the newly-reported chronic HB rate per 100,000 decreased by 66 % from 17.7 to 6.1 (p < 0.0001), with a reduction of 92 % (2.4 to 0.2;p < 0.001) in children ≤9 yoa and 83 % (7.2 to 1.2;*p* = 0.003) in those 10–19 yoa. The incidence of unspecified HB cases did not decrease significantly overall (5.9 vs. 5.4; *p* = 0.24), in children ≤ 9 yoa (0.3 vs. 0.2;*p* = 0.70) or 10–19 yoa (1.6 vs. 1.5;*p* = 0.45).

Overall, 91 % of cases ≤19 yoa were immigrants likely infected before arrival in Canada. Among those ≤9 yoa, there were 9 acute-HB case reports between 2005 and 2013, of whom 8 were not preventable by infant immunization.

**Conclusions:**

Two decades of school-based immunization coupled with prenatal screening achieved striking reduction in disease burden in the low-endemicity province of Quebec, Canada. The oldest cohorts targeted by catch-up campaigns are now beyond the average age at childbirth so that neo-natal transmission and the potential incremental benefit of infant immunization will likely further diminish.

## Background

While most countries have universal hepatitis B (HB) immunization programs targeting infants, eight of the 10 provinces of Canada initially opted for school-based pre-adolescent or adolescent HB immunization programs [1, 2]. In the province of Quebec (population: ~8.2million; birth cohort ~85,000/year), the publicly-funded HB prevention program commenced with immunization of high-risk individuals in 1983 (including infants in households with known HB carriers), was expanded to include routine pre-natal HBsAg screening in 1989, and further expanded to provide universal grade 4 elementary school-based HB immunization to pre-adolescents 8–10 years of age (yoa) in 1994 [2, 3]. A catch-up HB immunization campaign for high school students was conducted in 1999 [2]. Between 1994 and 2007, students were immunized with a three-dose schedule (0, 1, 6 months) with Engerix-B or Recombivax-HB at pediatric dosage and since 2008 a two-dose schedule of Twinrix-Junior (at 0, 6 months) has been used [4]. This school-based HB immunization program in Quebec has steadily delivered vaccine to ~85 % of students annually and in combination with the catch-up campaign, has provided protection to the vast majority of Quebec students born since 1983 [3].

Evaluation of the Quebec school-based HB immunization program one decade after its implementation showed dramatic effects: reported incidence of acute cases in Quebec decreased by 79 % overall and by 91 % in pre-adolescents and adolescents 10–19 yoa [5]. These trends were similar to those observed in another large Canadian province with an adolescent program [6]. While such early program evaluations showed striking population benefit, it was nevertheless reasoned that a universal infant HB immunization program could prevent residual cases in young children ≤9 yoa, particularly since infants are at highest risk of chronic-HB infection and acute-HB in young children may otherwise be missed because more often asymptomatic [7]. Further, routine three-dose immunization coverage with pediatric pentavalent vaccine (DTaP-Polio-Hib) exceeds 95 % so that switching to the available hexavalent vaccine additionally inclusive of HB could improve coverage and reduce injections related to the school program. Here, we report follow-up analyses using notifiable disease reports to assess potential epidemiologic benefit of a universal infant immunization program after two decades of a pre-adolescent school-based program in Quebec, Canada.

## Methods

The Quebec provincial registry of notifiable diseases was used to identify confirmed HB cases reported between 1990 and 2013. As per the Canadian national case definition [8], acute HB cases were defined as patients test positive for HBsAg or anti-HBcIgM in the context of compatible clinical history or probable exposure, or an HBsAg-positive newborn of an HBsAg-positive mother. Chronic HB cases were defined as patients with HBsAg or viral DNA detected at an interval of at least 6 months or detection of HBsAg in the absence of anti-HBcIgM and no acute-HB clinical symptoms. Unspecified cases were HBsAg or viral DNA positive individuals for which available information was insufficient to classify them as either acute or chronic. Clinical and demographic information on cases was retrieved from the standard questionnaires used by local public health units to investigate reported HB cases. This surveillance study using denominalized information was legally mandated by the provincial officer of public health of Quebec according to the Public Health Act and research ethics committee approval was not required. Information on epidemiology of hepatitis B in the province of Quebec for the period of 2000–2010 is also available at: http://www.inspq.qc.ca/pdf/publications/1847_Maladies_Vaccination_Declaration_Obligatoire.pdf.

All statistical analyses were performed using the Statistical Analysis System (SAS Institute Inc, Cary, NC, version 9.4). Chi-square tests were used for the assessment of HB trends from 1990 to 2013 and differences were considered significant at p<0.05. The hepatitis B incidence rates and the rates of potentially preventable cases of disease were calculated by dividing the number of new cases by the population by age group throughout each year, expressed as case reports per 100,000 population.

## Results

### Notifiable disease trends: acute and chronic HB report rates, 1990–2013

**Fig. 1:**
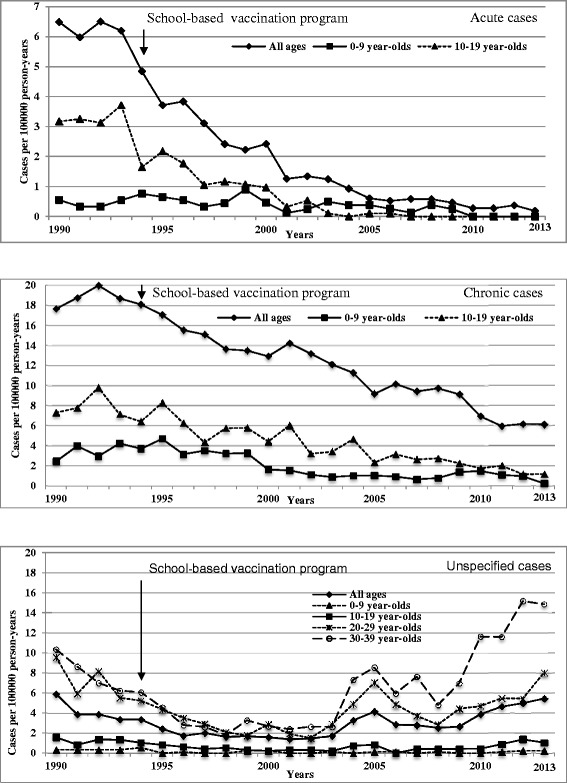
Reported rate per 100 000 person-years of acute, chronic and unspecified hepatitis B cases

Between 1990–2013, the overall incidence of acute-HB cases per 100,000 decreased by 97 % from 6.5 to 0.2 (p<0.0001), (Fig. 1). In children ≤9 yoa, the acute-HB incidence decreased from 0.6 in 1990 to no cases since 2010 (p < 0.0001). In those 10–19 yoa, the incidence of 3.2 in 1990 also decreased to zero since 2007 (p < 0.0001). Finally, in adults 20–29 yoa, the incidence of 15 per 100,000 in 1990, formerly the highest of all age groups, also fell to zero in 2013 (p < 0.0001).

During the same period, the overall rate of newly-reported chronic-HB cases per 100,000 decreased by 66 % from 17.7 to 6.1 (p < 0.0001), with a reduction of 92 % (2.4 to 0.2;p < 0.001) in children ≤ 9 yoa and 83 % (7.2 to 1.2;*p* = 0.003) in those 10–19 yoa. The rate of unspecified case reports showed an initial downward then upward trend during the study period. However, rates were low and stable throughout in children ≤ 9 yoa (0.3 vs. 0.2;*p* = 0.70) and 10–19 yoa (1.6 vs. 1.5;*p* = 0.45) old (Fig. 1). The upward trend in unspecified case reports since 2004 was among adults ≥20 yoa and primarily 30–39 yoa (Fig. 1).

### Acute-HB case reports – potential for prevention through infant immunization

Among children ≤9 yoa, there were 9 acute-HB case reports during the 2005–2013 period, all before 2010 and of whom 8 would not have been preventable by routine universal infant immunization. Five children had been adopted from HB-endemic countries and were already infected upon arrival in Canada. Three children were born in Quebec from HBsAg-positive mothers: two had received immunoglobulin at birth and 3 or 4 doses of vaccine according to the recommended schedule. The parents of the third child refused prenatal screening; the mother was identified to be HBsAg-positive 2 months post-delivery and the child was diagnosed with HB a few months after birth. The final acute HB case was born in another Canadian province and was diagnosed at preschool age. The mother was born in a highly-endemic country but her HBsAg status and the child’s age upon arrival in Quebec were unknown. Assuming that this latter case was preventable, then over and above the existing program, an infant immunization program could have spared 0.0249 acute-HB cases per 100,000 person-years (CI:0.0248-0.0251) in children ≤9 yoa between 2005 and 2009 and none between 2010 and 2013.

There were two acute-HB cases reported in individuals 10–19 yoa between 2005 and 2013; both occurred before 2008. The first was an unvaccinated homosexual boy born in Quebec; the second was a drug user with no information on country of birth or vaccination status. In addition to school-based HB vaccine-eligibility, these two individuals would have been eligible for immunization on the basis of high-risk behaviours but assuming infant immunization may have spared their infection, this would correspond to an additional preventable rate of 0.0654/100,000 person-years (CI:0.0651-0.0657) between 2005 and 2007 in that age group, and none between 2008 and 2013.

Overall, in addition to the existing program, an infant immunization program could have prevented 0.0335 (95 % CI: 0.0334-0.0336) acute-HB cases per 100,000 person-years between 2005 and 2009 in people 0–19 years old.

### Chronic-HB and unspecified cases – risk factors

With no acute-HB case reported in children ≤9 yoa since 2010, we limited the analysis of risk factors among chronic cases to the period spanning 2005–2009.

Among the 38 chronic-HB cases reported in children ≤9 yoa, 34 (89 %) were born outside Quebec, three were born in Quebec and one lacked place-of-birth information. The three Quebec-born children had known HBsAg-positive mothers, received adequate vaccination and immunoglobulin at birth and were not preventable. Among the 34 (89 %) immigrant cases, 13 were already infected upon arrival in Canada or diagnosed with chronic-HB during the first 12 months thereafter; 9 had unknown HBsAg status at arrival but were born from a known HBsAg-positive mother; and one was fully vaccinated before arrival. For the other 11 cases there was insufficient information to explain when and how they became infected.

In the 10–19 yoa group, there were 118 chronic-HB cases reported between 2005 and 2009. Of these, 9 cases were born in Quebec and 109 cases (92 %) were born outside Quebec (*n* = 84) or with unknown place of birth (*n* = 25). Among individuals born in Quebec, 2 were born to HBsAg-positive mothers, 2 had HBsAg carriers among their family members, 2 reported unsafe sex, 2 had parents born in an endemic country with unknown HB status and one did not report any risk factor. The two cases born to HBsAg-positive mothers were probably infected at birth and would likely not have been prevented by an infant program starting at 2 months of age. Among the other 109 chronic-HB cases, 80 (68 %) were born in a high-endemicity country, 2 had HBsAg carriers among their family members, 2 were born to an HBsAg-positive mother, and 1 reported sexual aggression as a potential cause of infection.

In addition, there were 15 case reports in people 10–19 yoa with unspecified acute or chronic HB status, 5 of whom were born in a high-endemicity country and another whose parents were born in an endemic country. For the other 9 unspecified cases, there was no information on country of birth or other risk factors.

Globally, among the 182 cases reported in those ≤19 yoa, 133 cases were born outside Quebec, 122 (91 %, CI:87 %-96 %) of whom were born in an endemic country.

## Discussion

This study using HBV notifiable disease statistics and case reports from a low-endemicity country, reveals the remarkable success of two decades of pre-adolescent immunization coupled with prenatal HBsAg screening in controlling HB not only in adolescents but also in children ≤9 yoa and adults 20–29 yoa. We measured 97 % reduction in acute-HB cases in the population overall since 1990, and an absence of acute cases in children ≤9 yoa since 2010, in adolescents 10–19 yoa since 2007, and in adults 20–29 yoa, formerly with the highest incidence, in 2013. In that context, it is difficult to assert that further program change is likely to bring important epidemiologic benefit.

During the study period, the rate of chronic-HB cases reported also dramatically decreased (by 92 % in children ≤9 yoa and 83 % in those 10–19 yoa). As the vast majority of chronic cases were born in highly-endemic countries, this large reduction is most likely attributable to universal infant immunization programs currently in place elsewhere in most high-endemicity countries, reducing chronic carriage in young immigrants and thereby also the risk of HB transmission during early childhood in Quebec. The potential for prevention of these chronic cases through infant immunization in Quebec would have been limited as most were born in endemic countries and likely infected before arriving in Quebec. Should some immigrant cases have acquired their infection after their arrival in Quebec, as they were up to 19 yoa, they could have been prevented only if an infant program had been in place since the early 1990s. Cases potentially preventable through infant immunization in 2014 are expected to be even lower recognizing no acute cases have been identified in children ≤9 yoa since 2010. Whether such further decrements in disease burden warrant program change ultimately depends upon operational, health care, societal and cost considerations but is less evident based on epidemiologic indicators.

This study has some limitations. It was based on passive surveillance reports of notifiable diseases with imperfect sensitivity likely to underestimate the true incidence of HB, particularly among young children with a greater frequency of mild or asymptomatic acute infection. While diagnostic approaches and clinician testing behaviours in response to patients presenting with acute viral hepatitis are unlikely to have changed substantially over the study period, we cannot exclude some variations in the detection of HB over time. Additionally, HB reporting has been mandatory for physicians and laboratories for several decades [5] and observed trends in acute cases are likely to be robust, reflecting extensive reduction in transmission and risk. While the unspecified case category may include acute cases, this would not change overall conclusions. The number of unspecified cases reported annually in the overall population varied from year-to-year without an evident or statistically significant trend. Unspecified cases were rare in those ≤19 yoa and the upward trend since 2004 was driven mainly by adults 30–39 yoa who were not vaccine-eligible by age. Finally, these results were obtained in a low-endemicity country and should not be extrapolated to moderate- or high-endemicity areas or those without HBsAg prenatal screening. However, as 18 % of the Quebec population is born outside Canada [9], including 54 % of immigrants from Asia, Africa or the Middle East [10], these results may nevertheless be relevant to other jurisdictions with low endemicity but a substantial proportion of immigrants from highly-endemic regions.

## Conclusion

In summary, two decades of pre-adolescent school-based immunization coupled with prenatal HBsAg screening has achieved striking reduction in population HB disease burden. Furthermore, the oldest cohorts targeted by catch-up campaigns are now beyond the average childbearing age (30 years) and the age at which the greatest number of births occur (28 years) in Quebec; as such, neo-natal transmission is anticipated to further diminish.

## Abbreviations

HB: Hepatitis B
HBsAg: Surface antigen of hepatitis B virus
DTaP-Polio-Hib: Diphtheria, tetanus, acellular pertussis, poliomyelitis and haemophilus influenzae type B vaccine
DNA: Deoxyribonucleic acid
95 % CI: 95 % confidence intervals
SAS: Statistical analysis system
